# Assessing the Risk of Hg Exposure Associated with Rice Consumption in a Typical City (Suzhou) in Eastern China

**DOI:** 10.3390/ijerph14050525

**Published:** 2017-05-12

**Authors:** Gang Wang, Yu Gong, Yi-Xin Zhu, Ai-Jun Miao, Liu-Yan Yang, Huan Zhong

**Affiliations:** 1 State Key Laboratory of Pollution Control and Resource Reuse, School of the Environment, Nanjing University, Nanjing 210023, China; gwang@189.cn (G.W.); gongyu111@hotmail.com (Y.G.); miaoaj@nju.edu.cn (A.-J.M.); 2 CQC Intime Testing Technology Co. Ltd., Suzhou 210023, China; cqc_zhuyx@126.com

**Keywords:** mercury, rice, food safety, dietary exposure

## Abstract

Recent studies have revealed that not only fish but also rice consumption may significantly contribute to human exposure to mercury (Hg) in Asian countries. It is therefore essential to assess dietary exposure to Hg in rice and its associated health risk. However, risk assessments of Hg in rice in non-contaminated areas are generally lacking in Asian countries. In the present study, Hg concentrations were measured in rice samples collected from markets and supermarkets in Suzhou, a typical city in Eastern China. In addition, the rice ingestion rates (IR) were assessed via a questionnaire-based survey of Suzhou residents. The data were then used to assess the risk of Hg exposure associated with rice consumption, by calculating the hazard quotient (HQ). Hg contents in rice samples were well below the national standard (20 μg/kg), ranging from 1.46 to 8.48 ng/g. They were also significantly (*p* > 0.05) independent of the area of production and place of purchase (markets vs. supermarkets in the different districts). Our results indicate a low risk of Hg exposure from rice in Suzhou (HQ: 0.005–0.05), despite the generally high personal IR (0.05–0.4 kg/day). The risk of Hg associated with rice consumption for Suzhou residents was not significantly affected by the age or sex of the consumer (*p* > 0.05). Overall, our results provide a study of human exposure to Hg in rice in Chinese cities not known to be contaminated with Hg. Future studies should examine Hg exposure in different areas in China and in potentially vulnerable major food types.

## 1. Introduction

Since the recognition of Minamata disease in 1956, mercury (Hg)-related food safety issues have been a matter of concern throughout the world, but especially in Asia. It is generally believed that fish/shellfish consumption is one of the major route of human exposure to Hg [1,2,3,4,5]. A significantly positive correlation (R = 0.401, *p* < 0.001, *n* = 174) was found between the consumption of fish and total Hg in the respondents [6]. Therefore, Hg contents in fish/shellfish and the associated health risk to consumers have been extensively studied [7,8,9]. Detrimental effects of Hg associated with fish/shellfish consumption have thus been reported in a number of studies, e.g., the European fish-eating cohorts under Public Health Impact of Low-level Mixed Element Exposure (PHIME), the Seychelles Child Development Study, and the New Zealand fish-eating cohort [10,11,12]. Recent studies indicate that rice consumption could also contribute significantly to human exposure to Hg [13,14]. In certain inland areas of China (e.g., Wanshan mining area), rice consumption was found to be responsible for the majority of human Hg exposure (as high as 42%) [15], due to the high consumption rates (600 g/day) of rice containing high Hg contents. The flooding conditions of rice paddies could facilitate Hg transformation to the highly bioaccumulative species methylmercury (MeHg), which accumulates in rice grains at elevated contents [16]. Zhang reported that MeHg has a bioconcentration factor which is on average >800 times higher than that of inorganic Hg (0.71 to 50 vs. 0.0014 to 0.51) [13]. Therefore, assessments of the risk of Hg exposure associated with rice consumption in Asian countries, where rice is the staple food for the majority of the respective populations, is essential.

In the last decade, the exposure of Asian populations to Hg in rice has been investigated in several studies, many of which focused on consumers in China [7,8,9,13,14]. In Guizhou Province, China, an area of intensive Hg mining, food (especially rice) rather than inhalation was shown to be the major route of human exposure to Hg. It is reported that food consumption accounts for more than 90% of the probable daily intake (PDI) of Total Hg. Specifically, rice accounted for 18.6–25.5%, while inhalation accounted for 3.8–6.8% [17], not to mention that rice can accumulate Hg from both atmosphere and soil [18]. In the same Hg mining region, MeHg levels in hair were shown to well correlate with dietary exposure to MeHg in rice, which could be explained by the overwhelming contribution of the rice consumption [19]. Those pioneering studies provided important insights into the risk associated with rice consumption in Asian populations and the need for comprehensive studies. However, most studies carried out thus far investigated Hg-contaminated areas, e.g., Hg mining areas, whereas little is known about exposure to rice Hg by the general population of China and of other Asian countries. This deficit of data may hinder accurate assessments of dietary exposure to Hg and the associated health risk; but these studies are important given that chronic exposure to low levels of Hg can induce nervous system dysfunction as well as renal, reproductive, immune, and cardiovascular damage and that the population of the non-contaminated area is much larger than its counterpart in Hg-contaminated areas [20,21]. Based on limited study, it has been found that most of the rice-consumption-related exposure of Hg is far below the Provisional Tolerable Weekly Intake (PTWI), as established by World Health Organization (WHO) in 1972 and adjusted by Joint Expert Committee on Food Additives (JECFA) from Food and Agriculture Organization (FAO)/WHO in 2010. Whether this finding can be extended to other areas needs to be further examined.

The aim of the present study is to evaluate the potential risk of Hg associated with rice consumption in a typical city in eastern China. Suzhou (Jiangsu Province) was selected principally because: (1) It is far away from Hg mining areas with its industry mainly made up of information technology, equipment manufacturing, textiles, tourism and is thus a representative of non-contaminated cities in China; (2) Suzhou has the second largest population and second highest gross domestic product (GDP) among cities in eastern China right after Shanghai.; (3) The rice ingestion rates (IRs) for consumers in Suzhou lie between those of consumers in northern China, where wheat is the main food, and those in southern China, where rice is the staple food [22]. Rice samples were collected from local markets and supermarkets in Suzhou and their Hg contents were quantified. In addition, a questionnaire-based survey was conducted to assess the IRs of consumers in Suzhou. The risk of Hg ingestion associated with rice consumption in city residents was then assessed by calculating the hazard quotient (HQ). Our results contribute to a better understanding of the risk of Hg for the general populations of Asian cities.

## 2. Materials and Methods

### 2.1. Sampling and Hg Analysis

**Figure 1:**
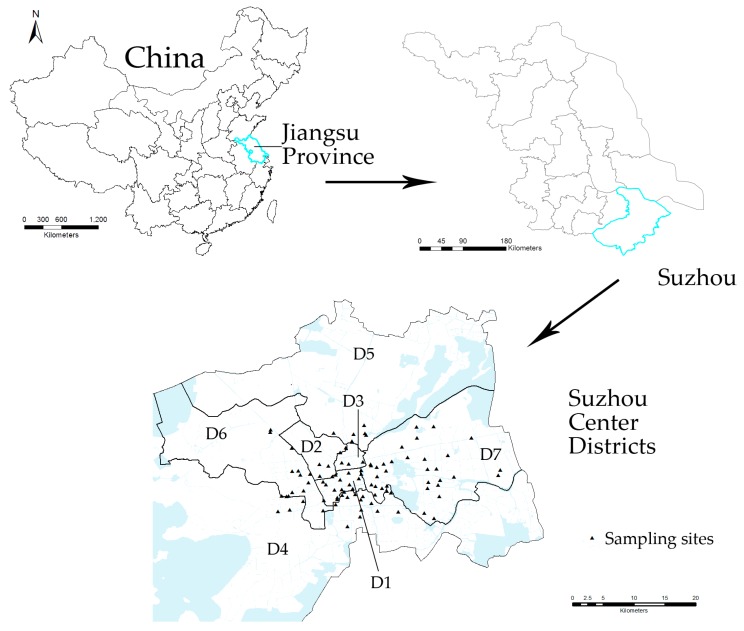
Sampling sites in Suzhou Center Districts, Jiangsu, China. D1: Canglang District, D2: Jingchang District, D3: Pingjiang District, D4: Wuzhong District, D5: Xiangcheng District, D6: Huqiu District, D7: Suzhou Industrial Park District.

Suzhou city, one of the major cities in Jiangsu Province, locates in the center of Yangtze River Delta. It is between 30°02′–32°02′ E and 119°55′–121°20′ N, bordering on Jiaxing and Huzhou of Zhejiang Province on the south, and Shanghai on the east. According to our preliminary survey, the major sources of rice for consumers in Suzhou are markets and supermarkets, which are primarily produced in northeastern China (NEC) and northern Jiangsu (NJS). Thus, the 177 rice samples analyzed in the present study were collected from local markets (3–27 per district) and supermarkets (2–7 per district) in Suzhou in November, 2016 (Figure 1). The locations of rice productions were recorded when collecting the rice samples (i.e., as NEC or NJS rice).

All rice samples were washed, freeze-dried, ground into powders, and sieved through 100-µm meshes. Considering the processing capacity (no more than 500 mg) of the instrument and its Hg detection limit of 0.2 ng/g, 100–180 mg rice powder was weighed in triplicates from each sample and their Hg contents measured using a DMA-80 (Milestone Inc., Sorisole, Italy) direct Hg analyzer, based on United States Environmental Protection Agency(USEPA) Method 7473 (1998). Certified reference material (GBW10043) with a total Hg content of 4.8 ± 0.8 ng/g and instrument blanks were used for quality control. The recoveries of Hg in the certified reference material ranged from 112% to 119%.

### 2.2. Questionnaire Survey

A questionnaire survey of randomly selected participants in different districts was conducted in November 2016. The questions in the questionnaire included: (1) the amount of rice consumed per meal (g/meal); (2) the frequency of rice consumption (meals/week); (3) the major source of rice purchase; and (4) personal information (sex, age and body weight). Of the 295 questionnaires distributed, 236 were completed and collected.

### 2.3. Health Risk Assessment

As there are not enough data to demonstrate the carcinogenicity of Hg [23], we use a method provided by USEPA to evaluate the potential health risk for non-carcinogenic effects of Hg exposure [24].

In this method, dietary Hg exposure through rice intake was indicated as the estimated daily intake (EDI, ng/kg body weight/day):

(1) $$\mathrm{EDI}=\frac{C\times \mathrm{IR}}{\mathrm{BW}}$$

where C is the Hg content (ng/g) in rice, IR is the rice ingestion rate (g/d), and BW is body weight, measured in kg.

The non-carcinogenic health risk HQ was used to assess the potential health risk of Hg as follows:

(2) $$\mathrm{HQ}=\frac{\mathrm{EDI}\times 7}{\mathrm{PTWI}}$$

where PTWI is the provisional tolerable weekly intake of Hg, which was adjusted from 5.0 μg/kg BW/week to 4.0 μg/kg BW/week by JECFA. A HQ value > 1 indicates a potential health risk due to Hg exposure from rice consumption.

### 2.4. Statistical Analysis

Significant differences (*p* < 0.05) were determined based on the results of a one-way analysis of variance (ANOVA) with post-hoc multiple comparisons (Tukey or Tamhane) (SPSS 11.0 by SPSS, Chicago, IL, USA). The normality (Kolmogorov-Smirnov and Shapiro-Wilk tests) and homogeneity of variance (Levene’s test) of the data were examined during the ANOVAs.

## 3. Results and Discussion

### 3.1. Mercury Contents in Rice in Suzhou City

The Hg contents of the rice samples from the different sources are listed in Table 1. They ranged from 1.46 to 8.48 ng/g (mean 3.83 ng/g). They were thus consistently lower than the national standard of 20 μg/kg (GB 2762-2012, China national food safety standard: Maximum limit of contaminants in food) [25] and within the ranges reported in non-contaminated areas of China. For example, in Zhejiang Province, Hg contents in rice were 5 ng/g ± 3 ng/g [14], in Jiangsu Province 5.7 ng/g [15], and in Chongqing city 1.9–4.2 ng/g [16]. As for the rice samples in other countries, their Hg content was 3.04 ng/g ± 2.07 ng/g in Europe [26] and 2.91 ng/g ± 0.86 ng/g in Republic of Korea [27]. These data imply that Hg contents in non-contaminated areas around the world are at the same order of magnitude.

**Figure 2:**
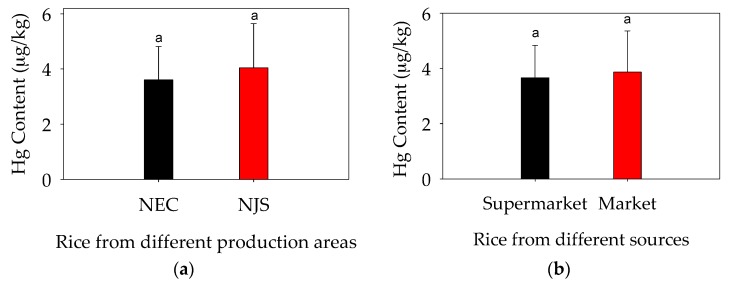
(**a**) Mercury content (mean ± standard deviation) in rice from different production areas, northeastern China (NEC) and northern Jiangsu (NJS); (**b**) Mercury content (mean ± standard deviation) in rice from different sources. Letter a in the figure indicate significant difference (*p* < 0.05).

When comparing the rice from different sources, Hg levels in rice from northeastern China (NEC: 1.67–8.31 ng/g, averagely 3.61 ng/g) and from northern Jiangsu (NJS) (1.46–8.48 ng/g, averagely 4.04 ng/g; Figure 2a) were comparable (*p* > 0.05) to each other. This finding is consistent with the generally low Hg contents in NEC and NJS soils: The average content of Hg in NJS topsoil is 0.1 mg/kg, and it is even lower for the soils of NEC, e.g., 0.064 mg/kg in Liaoning Province [28], 0.031 mg/kg in Heilongjiang Province [29], and 0.04 mg/kg for Jilin Province [30]. These values are much lower than the values stated in the national guideline for farmland (0.25 mg/kg when soil pH ≤ 5.5, 0.35 mg/kg when 5.5 < pH ≤ 6.5, 0.70 mk/kg when 6.5 < pH ≤ 7.5, 1.5 mg/kg when pH > 7.5, Environmental quality standards for soils, Ministry of Environmental Protection of China, GB 15618-2008) [31]. By contrast, the Hg content (11 mg/kg ± 1.9 mg/kg) in soils from Gouxi, Guizhou Province, was much higher than the national standard and thus the Hg content in the rice from these areas was in the range of 120 ng/g ± 33 ng/g with MeHg being the major Hg species [32]. Moreover, Hg levels were comparable in rice purchased from markets (1.64–8.48 ng/g, average 3.87 ng/g) and supermarkets (1.46–6.48 ng/g, average 3.66 ng/g; Figure 2b), because the sources of the rice were probably the same or similar (NEC and NJS). Thus, at least in Suzhou, where the rice was purchased (in markets or supermarkets), the sources of the rice sold in the city did not differ in their contributions to the risk of Hg exposure associated with rice consumption. Nevertheless, this may not be always the case in other non-contaminated areas.

### 3.2. Results of the Questionnaire Survey

The results of the 236 completed questionnaires are summarized in Table 2. Among the respondents, 58.5% were male and 41.5% female. According to the recommendations of the WHO, three age groups were defined in the present study: members of group I (*n* = 186) were 18–44 years old, those in group II (*n* = 40) 45–59 years old, and those in group III (*n* = 10) 60–74 years old. Rice was the staple food for 39%, wheat for 11%, and both for 50% of the respondents in Suzhou. The high percentage of Suzhou residents for whom rice is a staple food demonstrates the importance of assessing the risk of Hg exposure via rice in China. As determined in our preliminary study, markets (42%) and supermarkets (52%) were the major sites of rice purchase.

The IR for respondents in Suzhou ranged from 0.05 to 0.4 kg/day. The average of 0.2 kg/day was comparable to that reported for Jiangsu residents (0.21 kg/day, [33]). The IR of Suzhou residents was generally much higher than that of residents in northern provinces, where wheat rather than rice is preferentially consumed. For example, in Liaoning Province the annual IR is 54.4 kg (i.e., 149 g/day) [34]. According to a national survey, average rice consumption in northern China (e.g., 130.3 g/day in Heilongjiang Province, 118.3 g/day in Hebei Province, 53.9 g/day in Shanxi Province) is much less than southern China (e.g., 282.4 g/day in Fujian Province, 308.2 g/day in Sichuan Province, 260 g/day in Guangxi Province) [35]. In comparison, Americans consume 0.5 cup of cooked rice each day [36], that is about 28.3 g/day. Consumption of rice per capita for Bangladeshis in UK was 251 g/day, 30 times more than that of White Caucasians in UK [37]. Thus, it is evident that the IR for residents in Suzhou is a representative data for Asians, especially Southern Chinese.

**Figure 3:**
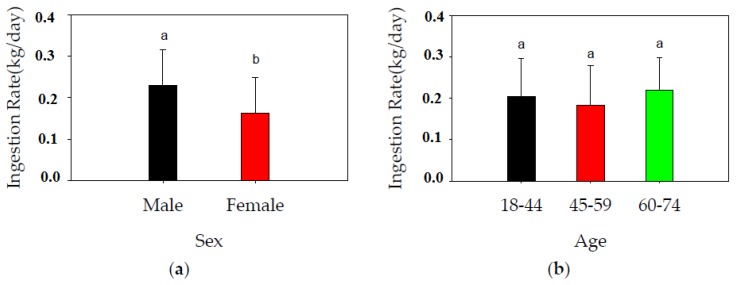
(**a**) Rice ingestion rates (mean ± standard deviation) according to the sex of the study participants; (**b**) Rice ingestion rates (mean ± standard deviation) according to the age of the study participants. Data of both male and female responses combined. Different letters a, b in the figure indicate significant difference (*p* < 0.05).

The IRs for male and female respondents were 0.23 ± 0.09 and 0.16 ± 0.09 kg/day, respectively. The difference was significant (*p* < 0.05) (Figure 3a) and in agreement with the rates reported elsewhere in China as well as other parts of the world. In Guangdong Province, average rice consumption by males is 10.1 kg/month, and that of females 8.8 kg/month [38]. In a cadmium-polluted area in China, individuals ranging in age from 20 to 59 years had a sex-related difference in rice consumption. In our study herein, males consumed 449.6 g rice/day, and females 351.4 g/day [39]. Age had only minor effects on the IR (*p* > 0.05), averaging 0.2 ± 0.09 (0.05–0.4) kg/day, 0.18 ± 0.1 (0.05–0.4) kg/day, and 0.22 ± 0.079 (0.1–0.3) kg/day for age groups I, II, and III, respectively (Figure 3b). This is consistent with rice being the staple food for people of different ages in eastern China. However, the result is based on questionnaires that excluded the underage group. It might be different if children were involved, as investigations showed that IR for adults is almost double (389.2 g/day vs. 198.4 g/day) [40] or even triple (324.4 g/day vs. 112.4 g/day) [41] that of children.

### 3.3. Risk Assessment of Hg in Rice for Suzhou Residents

The HQ, as obtained in the present study, ranged from 0.005 to 0.05 (mean of 0.02) and indicated a generally low risk of Hg exposure via rice consumption by Suzhou residents. Compared to the HQ related to fish consumption, the HQ of a health risk for Chinese residents due to Hg in rice has been less well documented. Rather, most studies have examined the risk posed by fish even though rice is the major source of dietary Hg exposure. Nonetheless, a comparison of our results with the limited number of HQ values reported in the literatures indicated that the HQ values in Suzhou were comparable to or lower than those reported from other non-contaminated areas of China. For example, the HQs of Zhejiang Province and Changshu city residents were 0.042 and 0.143, respectively [22,42]. By contrast, the values for Suzhou residents were lower than those documented for residents of Hg-contaminated areas in China. For example, in a zinc-lead mining area located in Hunan Province, the HQ was 0.70 [43], and for residents living near a mercury mine in Guizhou Province it was 1.7. In those studies, the Hg content in rice from the contaminated area in Hunan Province was 0.07 mg/kg and the IR was 0.43 kg/day, much higher than the corresponding values in our study. For residents of Guizhou Province, the IR was 0.39 kg/day; that study also reported a MeHg content in hair of 0.13 mg/kg. The results provide evidence of the potential health threat of Hg-contaminated rice [44] and the need to take into account both the IR and Hg content.

**Figure 4:**
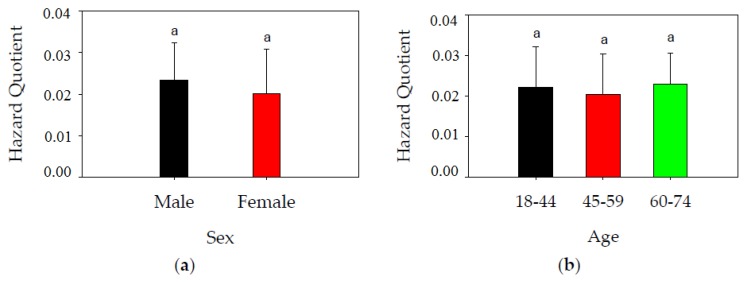
(**a**) Estimated risks (mean ± standard deviation) of Hg exposure in rice with respect to the sex of consumers; (**b**) Estimated risks (mean ± standard deviation) of Hg exposure in rice with respect to age of consumers. Data of both male and female responses combined. Letter a in the figure indicate significant difference (*p* < 0.05).

In the present study, the health risk related to Hg-contaminated rice was compared in the participants with respect to health and age. For age groups I, II and III, the HQ values were 0.022, 0.020 and 0.023, respectively; the differences were not significant (*p* > 0.05) (Figure 4a). This could be attributed to the less variable IR for consumers in different age groups. Similarly, comparable HQ values were found for male and female residents of Suzhou (*p* > 0.05), despite the higher IR for males (Figure 4b). This was mainly because of the generally lower body weight of female than male consumers. In addition, neither the place of purchase (98 markets vs. 123 supermarkets) nor the source of the rice (87 NEC, 90 NJS) significantly affected the HQ of Hg in rice. This observation can be explained by the comparable levels of Hg in rice among these categories. Although no significant differences between sexes was observed herein, there is a case [45] suggesting that women have a longer half-time of Hg in kidneys than men. This difference may lead to different risks for the two populations, which cannot be ignored and needs to be further examined. In contrast to HQ related to rice consumption, HQ related to fish consumption varies greatly between individuals as a result of their different habit or frequency of fish consumption. For instance, people who live near the sea eat fish more frequently and thus their HQ related to fish consumption was much higher than those far away from the sea [46]. For some particular individuals such as fishermen, HQ related to fish consumption is almost 3 times higher than that from rice (74%:23%) [47]. This is not only due to the higher content of Hg in fish but also because of the higher proportion of MeHg in fish (97.6%) as compared to rice (87.1%) [46]. However, it should be noted that differences in beneficial constituents in rice and fish should also be considered when comparing risks of Hg associated with rice and fish consumption. For instance, relatively high levels of long-chain omega-3 fatty acids were commonly found in fish, which may partly counteract the toxic effects of Hg in fish [48,49,50]. Therefore, the same dosage of Hg in fish and rice may not result in the same risk for human consumption, which should be considered in future studies.

**Figure 5:**
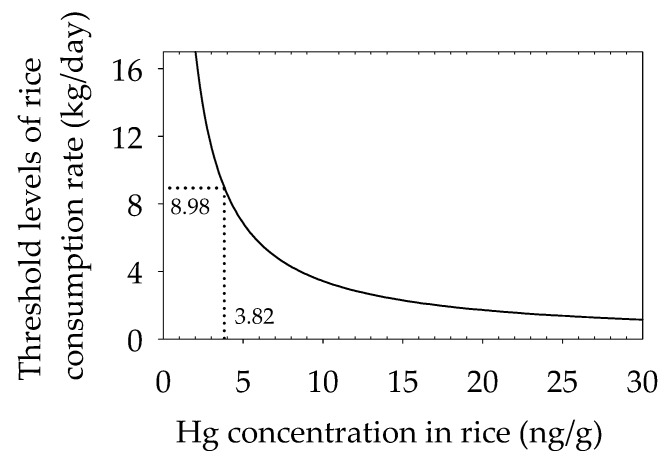
Threshold levels of consumption rate in accordance with the Hg concentration in rice.

Based on risk calculation, consumption advisory for Suzhou residents was provided (Figure 5): Threshold level of consumption rate for Suzhou residents is 8.98 kg per day for people with an average bodyweight of 60 kg, when Hg concentration in rice is at the average level (averagely 3.82 ng/g in this study). Considering the documented daily consumption rate of rice (0.21 kg per day for Suzhou residents [33]), our results may clearly suggest that risk of Hg associated with rice consumption is generally low for consumers in Suzhou city.

## 4. Conclusions

The results of the present study clearly demonstrated the low risk of Hg exposure associated with rice consumption by consumers in Suzhou city and may be representative of other cities in eastern China. Neither the place of purchase nor the source of the rice, nor the sex and age of consumers was associated with a health risk posed by the potential Hg contamination of rice. Future studies should compare the Hg levels in rice and fish, the two major sources of dietary Hg exposure in China, and their effects on human health. The findings will contribute to a better understanding of the risk of Hg in food for the general population in other non-contaminated areas of China.

## Figures and Tables

**Table 1:** Hg content in rice of different sources (NEC: northeastern China; NJS: northern Jiangsu) from different districts of Suzhou city.

District	Sampling Site Number	NEC (ng/g)	NJS (ng/g)	Sampling Site Number	NEC (ng/g)	NJS (ng/g)	Sampling Site Number	NEC (ng/g)	NJS (ng/g)
D1 (Canglang District)	S16	3.03	1.67	S19	1.94	2.81	S22	3.3	N.A.
	S23	4.08	5.26	S24	4.7	N.A.	S30	3.28	3.41
	S31	2.71	N.A.	S38	4.03	N.A.	S41	2.51	4.81
	S51	2.54	1.86						
D2 (Jinchang District)	S10	3.34	3.22	S11	8.31	3.65	S17	N.A.	3.41
	S18	3.95	8.48	S21	2.02	1.64	S25	N.A.	2.44
D3 (Pingjiang District)	S26	1.98	2.05	S28	2.7	3.79	S32	2.89	5.63
	S34	4.7	4.81	S35	2.98	3.78	S36	4.62	3.56
	S44	3.47	2.65	S45	4.3	N.A.	S48	3.31	2.98
D4 (Wuzhong District)	S1	2.82	3.91	S33	3.36	3.75	S39	3.44	3.93
	S42	3.77	4.69	S43	4.72	3.27	S46	3.82	5.57
	S47	3.13	3.84	S53	4.87	3	S58	2.55	2.1
	S63	6.84	2.58	S64	3	4.22	S67	4.18	3.2
	S92	2.73	2.13	S93	4.12	5	S94	2.43	4.42
D5 (Xiangcheng District)	S37	2.97	6.01	S96	5.53	2.74	S97	3.03	4.06
	S98	4.66	5.23	S100	5.67	2.1			
D6 (Huqiu District)	S2	4	4.27	S3	3.07	3.83	S4	3.79	2.5
	S5	3.34	4.01	S6	N.A.	4.21	S7	5.67	3.82
	S8	2.49	2.13	S9	2.48	4.02	S13	4.31	8.05
	S15	2.67	2.31	S86	3.52	5.16	S87	3.71	6.92
	S89	2.75	1.46	S90	2.87	7.63	S91	5.15	3.96
D7 (Suzhou Industrial Park District)	S49	2.49	5.75	S50	2.89	5.07	S55	4.25	2.57
	S56	3.16	6.42	S59	4.85	4.78	S60	3.45	3.31
	S61	4.36	5.16	S62	N.A.	2.98	S65	3.02	3.13
	S69	3.59	2.92	S71	3.7	4.58	S73	1.85	7.66
	S74	2.7	4.16	S75	3.71	6.07	S76	5.32	2.21
	S78	2.37	1.82	S79	3.66	2.51	S80	N.A.	1.7
	S82	N.A.	5.37	S83	1.67	5.19	S84	N.A.	2.84
	S85	4.12	4.22	S101	2.41	6.94	S102	4.97	6.25
	S103	3.92	3.7	S104	3.39	4.65			

N.A. Not available.

**Table 2:** Summary of the responses to the questionnaire survey in Suzhou city.

**Sex**	Male	Female			
	58.5%	41.5%			
Age *	Age < 30	30 ≤ Age < 40	40 ≤ Age < 50	50 ≤ Age	
	44.1%	27.1%	13.1%	15.7%	
Staple Food *	Rice	Wheat	Both	Other	
	38.6%	11.4%	50.0%	0.0%	
Source *	Supermarket	Market	Home	Canteen	Other
	52.1% **	41.5% **	4.7% **	28.0% **	2.1% **

* Data of both male and female responses combined; ** Multiple choices were allowed.
